# Care interruptions and mortality among adults in Europe and North America

**DOI:** 10.1097/QAD.0000000000003924

**Published:** 2024-05-14

**Authors:** Adam Trickey, Lei Zhang, Christopher T. Rentsch, Nikos Pantazis, Rebeca Izquierdo, Andrea Antinori, Gisela Leierer, Greer Burkholder, Matthias Cavassini, Jorge Palacio-Vieira, M. John Gill, Ramon Teira, Christoph Stephan, Niels Obel, Jorg-Janne Vehreschild, Timothy R. Sterling, Marc Van Der Valk, Fabrice Bonnet, Heidi M. Crane, Michael J. Silverberg, Suzanne M. Ingle, Jonathan A.C. Sterne

**Affiliations:** a Population Health Sciences, University of Bristol, UK; b School of Public Finance and Management, Yunnan University of Finance and Economics, China; c Yale School of Medicine, New Haven, CT, USA; London School of Hygiene & Tropical Medicine, London, UK; d Department of Hygiene, Epidemiology and Medical Statistics, Medical School, National and Kapodistrian University of Athens, Athens, Greece; e National Center for Epidemiology, Instituto de Salud Carlos III, Madrid, Spain; Centre of Biomedical Research for Infectious Diseases (CIBERINFEC), Carlos III Health Institute, Madrid, Spain; f National Institute for Infectious Diseases Lazzaro Spallanzani IRCCS, Rome, Italy; g Department of Dermatology and Venereology, Medical University of Innsbruck, Innsbruck, Austria; h Division of Infectious Diseases, University of Alabama at Birmingham, Birmingham, Alabama, USA; i Infectious Diseases Service, Lausanne University Hospital and University of Lausanne, Lausanne, Switzerland; j Centre for Epidemiological Studies on HIV/AIDS and STI of Catalonia (CEEISCAT); k Dept of Medicine, University of Calgary, Alberta, Canada; l Servicio de Medicina Interna, Hospital Universitario de Sierrallana, Torrelavega, Cantabria, Spain; m Department of Internal Medicine, Infectious Diseases, University Hospital Frankfurt, Frankfurt, Germany; n Department of Infectious Diseases, Copenhagen University Hospital, Rigshospitalet, Copenhagen, Denmark; o Department I for Internal Medicine, Faculty of Medicine and University Hospital Cologne, University of Cologne, Cologne, Germany; p Division of Infectious Diseases, Department of Medicine, Vanderbilt University School of Medicine, Nashville, TN, USA; q Stichting HIV Monitoring, Amsterdam, the Netherlands. Amsterdam University Medical Centers, Dept of Infectious diseases, University of Amsterdam, Amsterdam Institute for Infection and Immunity, Amsterdam, The Netherlands; r Université de Bordeaux, INSERM U1219, Bordeaux Population Health and CHU de Bordeaux, Service de Médecine Interne et Maladies Infectieuses, Hôpital Saint-André, Bordeaux, France; s Department of Medicine, University of Washington, Seattle, WA; t Kaiser Permanente Northern California, Oakland, CA, USA.

**Keywords:** adherence, antiretroviral therapy, mortality, North America, treatment gap, Western Europe

## Abstract

**Objective:**

Interruptions in care of people with HIV (PWH) on antiretroviral therapy (ART) are associated with adverse outcomes, but most studies have relied on composite outcomes. We investigated whether mortality risk following care interruptions differed from mortality risk after first starting ART.

**Design:**

Collaboration of 18 European and North American HIV observational cohort studies of adults with HIV starting ART between 2004 and 2019.

**Methods:**

Care interruptions were defined as gaps in contact of ≥365 days, with a subsequent return to care (distinct from loss to follow-up), or ≥270 days and ≥545 days in sensitivity analyses. Follow-up time was allocated to no/preinterruption or postinterruption follow-up groups. We used Cox regression to compare hazards of mortality between care interruption groups, adjusting for time-updated demographic and clinical characteristics and biomarkers upon ART initiation or re-initiation of care.

**Results:**

Of 89 197 PWH, 83.4% were male and median age at ART start was 39 years [interquartile range (IQR): 31–48)]. 8654 PWH (9.7%) had ≥1 care interruption; 10 913 episodes of follow-up following a care interruption were included. There were 6104 deaths in 536 334 person-years, a crude mortality rate of 11.4 [95% confidence interval (CI): 11.1–11.7] per 1000 person-years. The adjusted mortality hazard ratio (HR) for the postinterruption group was 1.72 (95% CI: 1.57–1.88) compared with the no/preinterruption group. Results were robust to sensitivity analyses assuming ≥270-day (HR 1.49, 95% CI: 1.40–1.60) and ≥545-day (HR 1.67, 95% CI: 1.48–1.88) interruptions.

**Conclusions:**

Mortality was higher among PWH reinitiating care following an interruption, compared with when PWH initially start ART, indicating the importance of uninterrupted care.

## Introduction

For people with HIV (PWH), antiretroviral therapy (ART) has substantially reduced the risk of mortality [1,2], leading to increased life expectancies [3,4]. The World Health Organization recommends that all PWH start ART when diagnosed, regardless of their HIV-related biomarkers such as CD4^+^ cell counts and HIV-1 RNA viral loads [5]. Globally, 76% of the 39 million PWH were estimated to be on ART in 2022 [6]. Although adherence to ART is key to maintaining a low risk of mortality [7], interruptions are common and occur for a variety of reasons, including treatment-related adverse effects and lifestyle factors [8].

Interruptions in HIV care are strongly associated with emergent viral resistance, AIDS-defining events, and mortality [8]. Key mathematical models of HIV epidemics, including those used by UNAIDS [9], account for CD4^+^ cell count, but assume that mortality rates are the same among PWH who resume care after an interruption as those starting ART for the first time, but there is considerable uncertainty around this assumption and how to better parameterize these models. Studies in Europe and North America comparing outcomes for PWH restarting care with PWH initiating ART for the first time have, due to a lack of mortality events, mostly used composite outcomes such as AIDS and/or death [10–13]. This does not allow for robust estimation of mortality rates.

We aimed to investigate the rates and predictors of interruptions from care among PWH on ART in Europe and North America, and whether mortality risk following restarting care after an interruption differed from mortality risk after initially starting ART.

## Methods

### Study design and population

Data were combined from 18 European and North American HIV cohort studies of PWH from the Antiretroviral Therapy Cohort Collaboration (ART-CC) [14], listed in the supplement. Ethics committees or institutional review boards approved the individual cohorts, which used standardized data collection methods, and regularly followed-up patients. Cohorts gathered information on mortality through linkage with vital statistics agencies and hospitals or physician report, and the active follow-up of participants.

Analyses were restricted to ART-naïve PWH starting ART regimens containing at least three drugs between 2004 and 2019. Eligible participants were aged ≥16 years old when starting ART and had no prior exposure to ART medications. Included participants had a CD4^+^ cell count and HIV-1 RNA viral load measurement within a window of 1 month before and 1 week after starting ART. We excluded PWH who started ART with an HIV-1 RNA viral load value of ≤400, because they may not have been ART-naïve.

### Interruptions to care

**Fig. 1:**
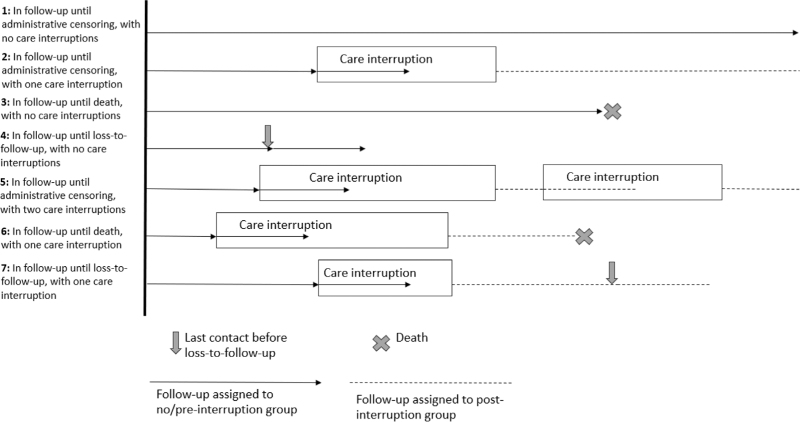
Illustration of how follow-up is partitioned to the no/preinterruption and postinterruption follow-up groups^∗^.

^∗^The first 365 days after the last contact before a care interruption or loss-to-follow-up is assigned to the prior follow-up period.

Recorded contact dates include dates of clinic visits, ART prescription/dispensing dates, and dates of laboratory tests; any of those listed in the HIV Cohorts Data Exchange Protocol (HICDEP) tblLAB: https://hicdep.org/Wiki/v/9/pt/4/Table/26/FieldID/305. We defined care interruptions as a gap in contact longer than 365 days, with a subsequent return to care. Care interruptions are thus distinct from loss to follow-up (LTFU), where there is no return to care. We assume that participants remain in care during the first 365 days of a care interruption. If someone is lost to follow-up, they are censored 365 days after the last recorded contact. Figure 1 illustrates how follow-up is partitioned to the no/preinterruption and postinterruption follow-up groups.

### Statistical analyses

Follow-up started at time of ART initiation. Participants were deemed to be under observation until the earliest of the date of death, 365 days after the last recorded contact (loss to follow-up), or date of administrative censoring (the last of which was February 29, 2020). To avoid under ascertainment of deaths, if somebody died after being lost to follow-up, then they would be no longer be considered lost and would be reincluded. Time on ART was allocated across two groups: “no previous interruption” (group A) and “postinterruption” (group B). Participants that had one or more care interruptions would contribute multiple follow-up periods to the analysis. Observation time after ART start and the first 365 days during the first care interruption was allocated to group A. If there was a care interruption then observation time after the first care interruption was allocated to group B, up until another care interruptions or the censoring date. Follow-up periods for participants who return to care with a suppressed viral load were excluded, due to uncertainty about whether an interruption in care had occurred.

We used Cox regression to compare hazards of mortality across the two care interruption groups, adjusting for time-updated characteristics and biomarkers upon ART initiation or re-initiation of care: age (16–24, 25–34, 35–44, 45–54, 55–64, ≥65), male or female sex at birth, CD4^+^ count (cells/mm^3^) (0–49, 50–99, 100–199, 200–349, 350–499, ≥500), year of ART initiation (2004–2007, 2008–2011, 2012–2015, 2016–2019), and method of HIV acquisition (sex between men, injecting drug use, heterosexual sex, and other/unknown). We stratified the baseline hazards by cohort. For determining CD4^+^ cell count and HIV-1 RNA viral loads upon re-initiation of care, we used window periods of 6 months prior to 30 days after the date of re-initiation. Where people were missing CD4^+^s cell count at re-initiation we assumed the CD4 count from the start of their prior initiation/re-initiation period, as these CD4^+^ cell count would likely rise whilst on ART and then fall when off ART. We performed several sensitivity analyses, details of which are listed in the supplementary materials.

We also used Cox regression to compare the hazard of first care interruption across baseline demographic and HIV-related factors: age, sex, CD4^+^ cell count, year of ART initiation, and method of HIV acquisition, again stratifying the baseline hazards by cohort.

To investigate the causes of mortality among people who had interruptions, we included cause of death data generated using similar methodology to our previous analyses [15,16]. Data from four cohorts where causes of death were unavailable were excluded. We used Cox regression to compare grouped cause-specific hazards of mortality across the two care interruption groups, adjusting for the same time-updated characteristics and biomarkers upon ART initiation or re-initiation of care as in the all-cause mortality analysis.

All statistical analyses were performed using STATA version 17 (STATA Corporation, College Station, Texas, USA).

## Results

In total, 89 187 PWH were included in the analysis. Table 1 shows the characteristics at ART start of the 80 533 PWH (90.3%) who never had a care interruption, and 8654 (9.7%) of PWH who had a care interruption. The median age of the overall sample was 39 years [interquartile range (IQR): 31–48], and 83.4% were male at birth. The median CD4^+^ cell count at ART initiation was 283 cells/mm^3^ (IQR: 146–421) among those who never had a care interruption, whilst the median ART start CD4^+^ cell count was 250 (IQR: 124–370) cells/mm^3^ for those who had an interruption. The distribution of the year of first starting ART differed between the interruption groups, with a lower percentage of PWH in the interruption group 281 (3.3%) having started ART 2016–2019 than in the no interruption (12545, 15.6%). A higher percentage of PWH that had care interruptions had acquired HIV through injecting drug use, 1398 (16.2%), than the no interruption group, 5006 (6.2%). Similarly, higher percentages of PWH in the interruption group, 2086 (24.1%), had an other/unknown HIV acquisition method than the PWH in the no interruption group, 14 632 (18.2%). There was a higher percentage of women in the interruption group (20.5%) than in the no interruption group (16.2%).

**Table 1:** Participant's characteristics at ART initiation and time-updated characteristics at the beginning of each follow-up period, stratified by ≥1 year interruption status.

Variable	Total cohort	No interruption	Interruption
	*N* (%) or median (IQR)	*N* (%) or median (IQR)	*N* (%) or median (IQR)
Number of participants	89187 (100.0%)†	80533 (90.3%)†	8654 (9.7%)†
Age (years)	39 (31–48)	40 (32–48)	38 (30–46)
ART initiation CD4^+^ cell count (cells/mm^3^)	280 (143–417)	283 (146–421)	250 (124–370)
Sex			
Female	14781 (16.6%)	13006 (16.2%)	1775 (20.5%)
Male	74406 (83.4%)	67527 (83.9%)	6879 (79.5%)
Year of first initiating ART			
2004–2007	20107 (22.5%)	16884 (21.0%)	3223 (37.2%)
2008–2011	27789 (31.2%)	24671 (30.6%)	3118 (36.0%)
2012–2015	28465 (31.9%)	26433 (32.8%)	2032 (23.5%)
2016–2019	12826 (14.4%)	12545 (15.6%)	281 (3.3%)
HIV acquisition method			
Sex between men	42222 (47.3%)	39436 (49.0%)	2786 (32.2%)
Injecting drug use	6404 (7.2%)	5006 (6.2%)	1398 (16.2%)
Heterosexual sex	23843 (26.7%)	21459 (26.7%)	2384 (27.6%)
Other/unknown	16718 (18.7%)	14632 (18.2%)	2086 (24.1%)
	All follow-up periods	No/preinterruption	Postinterruption
Number of follow-up periods	100100 (100.0%)†	89187 (89.1%)†	10913 (10.9%)†
Time-updated age (years)	40 (32–48)	39 (31–48)	43 (36–51)
Time-updated CD4^+^ cell count (cells/mm^3^)	282 (143–426)^a^	280 (143–417)	344 (144–595)^b^
0–49	11594 (11.6%)	10377 (11.6%)	1273 (11.7%)
50–99	7286 (7.3%)	6508 (7.3%)	811 (7.4%)
100–199	14741 (14.7%)	13185 (14.8%)	1599 (14.7%)
200–349	29768 (29.7%)	27243 (30.6%)	2575 (23.6%)
350–499	19292 (19.2%)	17454 (19.6%)	1788 (16.4%)
≥500	17579 (17.5%)	14420 (16.2%)	2867 (26.3%)

Column percentages are shown. †Row percentage. IQR, interquartile range.

a Time-updated values missing for 5628 (5.6%), so prior values used instead.

b Time-updated values missing for 5628 (51.6%), so prior values used instead.

For the main analysis with interruptions in care of a year or more defined as a care interruption, there were 100 100 follow-up periods included from the 89 187 PWH, with 8654 PWH having at least one care interruption. Of these 8654, 1782 had two interruptions, 383 had three interruptions, and 94 had four or more interruptions. With this definition of an interruption, the average length of time between the last contact date before the interruption and reinitiation of care was 527 days (IQR: 420–779). Each PWH contributed follow-up to the pre interruption group and there were 10 913 episodes of follow-up included following an interruption. Table 1 shows the age and CD4 counts time-updated to the beginning of each follow-up period. PWH were older at the start of the interruption episodes, 43 years (IQR: 36–51), than at ART start, 39 years (IQR: 31–48). The median CD4^+^ cell count at ART start was 280 cells/mm^3^ (IQR: 143–417), whilst it was 344 (IQR: 144–595) upon return to follow-up after an interruption where these data were available (for 51.6%). Table 1 also shows the CD4^+^ cell count categories used after assuming prior CD4^+^ values for PWH who returned to follow-up with missing CD4^+^ cell count data, with a higher percentage restarting follow-up after an interruption having CD4^+^ cell count of ≥500 (2867, 26.3%), than at ART initiation (14420, 16.2%). The percentage with the CD4^+^ cell count of 0–49 was similar in both groups, 10 377 (11.6%) after ART initiation, and 1273 (11.7%) after an interruption.

### Rates and predictors of first care interruption

Table 2 shows the crude rates per 1000 person-years of having a care interruption for ART start characteristics and the corresponding adjusted hazard ratios. From 509 060 years of follow-up, the overall rate of first interruption was 17.0 [95% confidence interval (95% CI): 16.6–17.3] per 1000 person-years. The median length of follow-up before a first interruption was 1956 days (IQR: 997–3197). Men had lower rates of care interruption than women, whilst age was the biggest predictor of care interruptions, with increasing age being associated with lower rates consistently across age-groups. PWH who had acquired HIV through injecting drug use, those who had acquired HIV through heterosexual sex and those with other/unknown HIV acquisition method had higher rates of care interruptions than PWH who had acquired HIV through sex between men.

**Table 2:** Crude rates and adjusted hazard ratios of the first care interruption of ≥1 year.

Variable	Person-years of observation	Care interruptions	Crude rate to first care interruption per 1000 person-years (95% CI)	Adjusted hazard ratio (95% CI)^a^
Overall	509060	8654	17.0 (16.6–17.3)	
Sex				
Female	88870	1775	20.0 (19.1–20.9)	1 (reference)
Male	420190	6879	16.4 (16.0–16.8)	0.92 (0.86–0.98)
Age at ART initiation (years)				
16–24	29082	710	24.4 (22.7–26.3)	1 (reference)
25–34	136517	2628	19.3 (18.5–20.0)	0.79 (0.72–0.85)
35–44	170577	2865	16.8 (16.2–17.4)	0.62 (0.57–0.68)
45–54	114444	1792	15.7 (14.9–16.4)	0.52 (0.48–0.57)
55–64	45948	532	11.6 (10.6–12.6)	0.38 (0.34–0.43)
≥65	12492	127	10.2 (8.5–12.1)	0.32 (0.27–0.39)
CD4^+^ cell count at ART initiation (cells/mm^3^)				
≥500	62428	1031	16.5 (15.5–17.6)	1 (reference)
350–499	92764	1410	15.2 (14.4–16.0)	0.91 (0.84–0.98)
200–349	172809	2835	16.4 (15.8–17.0)	0.98 (0.91–1.05)
100–199	82046	1573	19.2 (18.2–20.1)	1.12 (1.03–1.21)
50–99	38369	714	18.6 (17.3–20.0)	1.09 (0.99–1.20)
<50	60644	1091	18.0 (17.0–19.1)	1.03 (0.94–1.12)
HIV acquisition method				
Sex between men	240480	2786	11.6 (11.2–12.0)	1 (reference)
Injecting drug use	32975	1398	42.4 (40.2–44.7)	2.68 (2.50–2.86)
Heterosexual sex	141916	2384	16.8 (16.2–17.5)	1.39 (1.31–1.49)
Other/unknown	93689	2086	22.3 (21.3–23.2)	1.52 (1.38–1.68)

CI, confidence interval.

a Results are adjusted for all variables listed in the table, with stratification of hazards by cohort.

### Care interruptions and mortality risk

In the main analysis with care interruptions defined as gaps in care of at least 1 year, there were 6104 deaths in 536 334 person-years, a crude mortality rate of 11.4 (11.1–11.7) per 1000 person-years. Table 3 presents the crude mortality rates and adjusted mortality hazard ratios for characteristics time-updated for the beginning of each follow-up period. The crude mortality rate for the post interruption group was higher, 23.6 (95% CI: 21.8–25.5) per 1000 person-years, than the no/preinterruption group, 10.7 (95% CI: 10.5–11.0). The corresponding adjusted hazard ratio for the postinterruption group was 1.72 (95%CI: 1.57–1.88) compared with the no/preinterruption group. Mortality rates were higher for men than women, and for people who had acquired HIV through injecting drug use than through other methods. Mortality increased as CD4^+^ cell count decreased and age at the beginning of follow-up increased, whilst mortality was lower for follow-up beginning in later years.

**Table 3:** Crude mortality rates and adjusted mortality hazard ratios for ≥1 year interruption status, demographic characteristics, and HIV-related characteristics.

Variable	Person-years of observation	Deaths	Crude mortality rate per 1000 person-years (95% CI)	Adjusted hazard ratio (95% CI)^a^
Overall	536334	6104	11.4 (11.1–11.7)	
Interruption status				
No/preinterruption	509408	5469	10.7 (10.5–11.0)	1 (reference)
Postinterruption	26925	635	23.6 (21.8–25.5)	1.72 (1.57–1.88)
Sex				
Female	95080	794	8.4 (7.8–9.0)	1 (reference)
Male	441236	5310	12.0 (11.7–12.4)	1.23 (1.13–1.34)
Age at ART initiation or care re-initiation (years)				
16–24	29872	105	3.5 (2.9–4.3)	1 (reference)
25–34	142396	628	4.4 (4.1–4.8)	1.12 (0.91–1.38)
35–44	179795	1420	7.9 (7.5–8.3)	1.74 (1.42–2.12)
45–54	122421	1926	15.7 (15.0–16.5)	3.05 (2.50–3.71)
55–64	48713	1428	29.3 (27.8–30.9)	5.22 (4.26–6.38)
≥65	13137	597	45.4 (41.9–49.2)	8.44 (6.84–10.42)
CD4^+^ cell count at ART initiation or care re-initiation (cells/mm^3^)				
≥500	68974	357	5.2 (4.7–5.7)	1 (reference)
350–499	96991	564	5.8 (5.4–6.3)	1.13 (0.99–1.29)
200–349	179213	1523	8.5 (8.1–8.9)	1.47 (1.31–1.66)
100–199	86341	1219	14.1 (13.3–14.9)	2.11 (1.87–2.38)
50–99	40549	854	21.1 (19.7–22.5)	2.90 (2.56–3.29)
<50	64266	1587	24.7 (23.5–25.9)	3.31 (2.94–3.72)
Year of ART initiation or care re-initiation				
2004–2007	173378	2586	14.9 (14.4–15.5)	1 (reference)
2008–2011	198601	2125	10.7 (10.3–11.2)	0.77 (0.73–0.82)
2012–2015	134164	1157	8.6 (8.1–9.1)	0.59 (0.54–0.64)
2016–2019	30191	236	7.8 (6.9–8.9)	0.39 (0.34–0.45)
HIV acquisition method				
Sex between men	247746	1331	5.4 (5.1–5.7)	1 (reference)
Injecting drug use	37991	905	23.8 (22.3–25.5)	3.54 (3.23–3.89)
Heterosexual sex	149732	1310	8.8 (8.3–9.2)	1.32 (1.22–1.44)
Other/unknown	100865	2558	25.4 (24.4–26.4)	1.96 (1.74–2.20)

CI, confidence interval.

a Results are adjusted for all variables listed in the table, with stratification of hazards by cohort.

Cause-specific mortality rates were higher in the postinterruption group for each cause, except for suicides/accidents and liver-mortality, whilst confidence intervals were very wide for respiratory-related mortality (Table 4). AIDS-related mortality was higher in the postinterruption group, however, the biggest differences between the no/preinterruption and postinterruption follow-up groups were seen for non-AIDS infection mortality, followed by mortality due to non-AIDS, nonhepatitis malignancies, heart/vascular-related mortality, and mortality due to unknown causes.

**Table 4:** Cause-specific mortality rates in the no/preinterruption and postinterruption groups^a^.

	No/preinterruption			Post interruption			
Cause of mortality	Deaths	Crude MR	95% CI	Deaths	Crude MR	95% CI	aHR (95% CI)
AIDS	837	2.0	1.9–2.2	75	3.5	2.8–4.4	1.46 (1.13–1.88)
Heart/vascular	198	0.5	0.4–0.6	20	0.9	0.6–1.5	2.10 (1.25–3.52)
Liver	153	0.4	0.3–0.4	16	0.8	0.5–1.2	1.28 (0.73–2.24)
Non-AIDS infection	191	0.5	0.4–0.5	43	2.0	1.5–2.7	2.85 (1.95–4.17)
Non-AIDS, nonhep malig	486	1.2	1.1–1.3	38	1.8	1.3–2.5	2.16 (1.51–3.11)
Respiratory	86	0.2	0.2–0.3	10	0.5	0.3–0.9	1.88 (0.89–3.97)
Substance abuse	81	0.2	0.2–0.2	16	0.8	0.5–1.2	1.95 (1.06–3.60)
Suicide/accident	147	0.4	0.3–0.4	9	0.4	0.2–0.8	1.11 (0.54–2.26)
Other	335	0.8	0.7–0.9	46	2.2	1.6–2.9	1.70 (1.20–2.40)
Unknown	697	1.7	1.6–1.8	98	4.6	3.8–5.6	2.10 (1.65–2.66)
Total	3211	7.9	7.6–8.1	371	17.5	15.8–19.4	1.83 (1.63–2.06)

aHR, adjusted hazard ratio; CI, confidence interval; crude MR, crude mortality rate per 1000-person years; Non-AIDS, nonhep malig, non-AIDS, nonhepatitis malignancies.

a In a subset of cohorts with cause of death information available.

### Sensitivity analyses

**Fig. 2:**
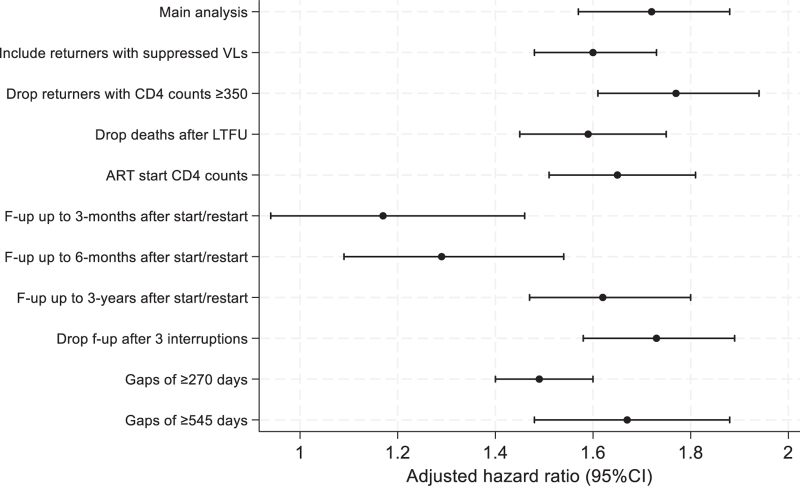
Sensitivity analyses of adjusted mortality hazard ratios∗ for interruption status.

ART, antiretroviral therapy; F-up, follow-up. ∗Adjusted for sex, age at ART initiation/re-initiation, CD4^+^ cell count at ART initiation/re-initiation, year of ART initiation/re-initiation, and HIV acquisition method, with hazards stratified by cohort.

Figure 2 and Table 1, Supplemental Digital Content present results of various sensitivity analyses. In all but one sensitivity analysis, mortality was elevated in the postinterruption follow-up group, compared with no/preinterruption. In the sensitivity analysis restricting follow-up to 3 months after ART initiation or re-engagement with care, the aHR for mortality in the postinterruption group, in which there were 105 deaths, was 1.17 (95% CI: 0.94–1.46). In that sensitivity analysis, the associations of time-updated age and CD4^+^ cell count with mortality were much stronger than in the main analysis; aHRs 20.9 (95% CI: 9.1–47.9) for being aged ≥65 vs. 16–24 years, and 16.2 (95% CI: 10.7–24.4) for having a CD4^+^ cell count <50 vs. ≥500 cells/mm^3^. In the sensitivity analysis where we categorized the number of interruptions (0, 1, 2, ≥3), the aHR for mortality was higher following a 2nd interruption, 2.20 (95% CI: 1.82–2.65) vs. 0 interruptions, than following a 1st interruption, 1.67 (95% CI: 1.51–1.83), whilst confidence intervals were wide for the ≥3 interruption group. When categorizing the length of the interruptions, we saw similar aHRs for each interruption length compared to no interruption: 365–547 days (aHR: 1.73 [1.54–1.93]), 548–729 days (aHR: 1.72 [1.43–2.06]), 730–1094 days (aHR: 1.65 [1.34–2.04]), and ≥1095 days (aHR: 1.81 [1.43–2.29]).

## Discussion

Our results indicate that the relative hazard of mortality is higher among PWH upon reinitiating care following a care interruption, compared with PWH starting ART for the first time; the adjusted hazard ratio for the postinterruption group was 1.72 (95% CI: 1.57–1.88) compared with the no/preinterruption group. These results were robust in various sensitivity analyses, except when limiting follow-up to just 3 months after ART initiation or re-engagement with care where there were far fewer deaths. The adjusted hazard ratio for mortality was higher following a 2nd interruption than following a 1st interruption, indicating a dose–response relationship. The adjusted cause-specific mortality hazard ratios indicated that the biggest difference between the two groups were in non-AIDS mortality, particularly non-AIDS infections, although AIDS-related mortality was also elevated. Age was the best predictor of care interruptions, with increasing age being associated with lower absolute rates and relative hazards consistently across age-groups, perhaps reflecting differing service delivery for older age groups [17] or differing lifestyle factors by age [18], whilst PWH who had acquired HIV through injecting drug use had higher rates of care interruptions than other HIV acquisition groups, perhaps due to high incarceration rates in this group [19].

### Comparison with other literature

Other studies have examined outcomes among PWH following an interruption in care or treatment. However, comparing these studies can be difficult due to different definitions of these interruptions and the statistical methods used. A systematic review of treatment interruptions, including studies from outside of Europe and North America, noted that they are common, and with a median duration of 150 days [8], although this study was published over a decade ago when HIV care was different. As we used a minimum of 365-day gaps to define a care interruption, our median duration of an interruption was much higher, 527 days. The systematic review found that the most frequently reported reasons for these interruptions were side-effects, adverse events, and drug toxicity [8]. Similar to the findings in our study, they found that younger age and injecting drug use were key risk factors for these interruptions [8]. In an analysis of the Italian ICONA Foundation Study cohort, which is part of the ART-CC, the authors found that younger age and injecting drug use were risk factors for being lost to care [20]. With interruptions defined using gaps of >18 months, PWH who then re-entered care were at higher risk than those who were continuously in care of a composite endpoint of death, AIDS-related infections, serious non-AIDS-related events, or hospitalization [20]. The Dat’AIDS cohort in France looked at care interruptions defined as intervals of >15 months and also found that younger patients were more likely to have interruptions [12]. They found that these interruptions were associated with increased odds of AIDS and, separately, death [12]. Another study from France defining interruptions using gaps of >12 months found that almost half of returning PWH had CD4^+^ cell count <200 or AIDS, and that those experiencing these interruptions were five times more likely to die [11]. In the CoRIS cohort in Spain, also part of the ART-CC, where interruptions were defined using gaps of >15 months, risk factors for interruption included younger age, lower educational level, having acquired HIV infection through injecting drug use or heterosexual intercourse, having been born outside of Spain, and CD4^+^ cell count >200 cell/μl, viral load <100 000 and co-infection with hepatitis C virus at enrolment [21]. In a study of the EuroSIDA cohort from 2007, when defining treatment interruptions using gaps of 3 months, they also found that a composite endpoint of AIDS events or death was more common in the group that had treatment interrupted, than in the group with no interruptions [22]. There has also been much research into treatment/care interruptions among PWH outside of Europe. For example, a study using data from a range of countries in East Africa showing that CD4 cell-count increases after restarting ART were slower than prior to the interruption [23], perhaps explaining the higher mortality rates postinterruption seen in our study and others.

### Strengths and limitations

The strengths of this study included the large sample size, geographical diversity, and representativeness of the PWH that were included for the regions they live. Therefore, our findings are likely generalizable to other similar concentrated epidemics. However, the cohorts are from various countries with different health systems, population characteristics, and migration patterns, so pooling such data may obscure within-country patterns. Specific centres with the included cohorts may have been undertaking different interventions to improve adherence to ART and reduce care interruptions, however, we were unable to account for this in our analysis. Additionally, adherence to ART will vary between PWH; having ART prescribed does not mean adherence will be good, even if someone turns up to their appointments. There may be some under ascertainment of mortality among the cohorts, however, most had linkage to either national or regional death registries, whilst the remainder had robust systems in place to determine mortality among PWH who disconnected from care [14,24]. Some PWH were missing data on CD4^+^ cell count when returning to care following an interruption, so time-updated CD4^+^ cell count upon return to care could not always be calculated and we had to instead make assumptions using their previous CD4^+^ cell count. We did not adjust the models for clinical stage (either AIDS or non-AIDS-related) as updates in clinical stage, such as AIDS-related events, would potentially have been missed during interruptions in care, and for non-AIDS-related conditions, there was variable reporting across the cohorts. There will likely have been unmeasured confounding, with the potential for the cause of the interruptions to have also been associated with mortality. Also, having a care interruption may be a predictor of future suboptimal adherence, which then leads to mortality – this difference between the no/preinterruption and postinterruption groups will not have been fully captured in the Cox models. It is difficult to define care interruptions, so we used three definitions (≥1 year, ≥270 days and ≥545 days) and used a sensitivity analysis to investigate our assumptions around PWH returning to care with suppressed to viral loads, as it is possible that PWH could temporarily transfer health provider. We did not have data on the reason for these care interruptions. Finally, the data used were from the ART-CC's 2019 data update from before the COVID-19 pandemic. The COVID-19 pandemic may have changed the epidemiology around care interruptions through increases in “telehealth” interventions and longer lengths of prescriptions in response to lockdowns and more restrictions regarding accessing care [25,26].

### Implications

Our results add further weight to the evidence that care interruptions for PWH on ART are associated with increased mortality. We showed that absolute rates and relative hazards of mortality following a care interruption were higher than after initially starting ART despite those returning to care, on average, having higher CD4^+^ cell count. Previous research has identified many reasons for care interruptions, including factors such as temporary migration, and, commonly, adverse effects from ART regimens [7,8]. Additionally, interventions have been developed to increase adherence to HIV care and reduce interruptions [27], such as community meetings [28], peer support [29], and counselling [30]. Care interruptions were common in this large population of PWH from a variety of countries and healthcare systems, indicating that scale-up of interventions to reduce care interruptions is required, as well as the development of additional interventions. Both AIDS-related mortality and a variety of non-AIDS-related mortality rates were higher in the post interruption group than in the no/preinterruption group, showing that the elevated mortality is potentially not due to a single mechanism. As the reasons for care interruptions are varied, there is also a need to identify the most likely reasons for a care interruption in certain settings and populations (for example, people who are likely to move away for work, or those with substance use issues) and which intervention is most likely to help in each instance. The results of this analysis should be used to parameterize mathematical models of HIV epidemics, including the Spectrum model used by UNAIDS [9], regarding the assumptions for mortality rates following care interruptions with similar concentrated epidemics. Adding the results of our analyses to such models should increase the accuracy of such models and improve the estimates produced for HIV policymakers in many settings. However, further analyses of care interruptions using data following the COVID-19 pandemic are likely required due to adaptions in care delivery that occurred during this time [26].

## Conclusions

The relative hazard of mortality was higher among PWH in Europe and North America upon reinitiating care following a care interruption, compared with PWH starting ART for the first time – these results were robust in various sensitivity analyses. The adjusted mortality hazard ratio was higher after a 2nd interruption than after a 1st interruption from care, indicating that the number of interruptions was also important. Both AIDS-related and non-AIDS-related mortality rates were elevated following a care interruption.

## Acknowledgements

We would like to thank our funders (US NIAAA) as well as all the patients and the clinical teams associated with the participating cohort studies.

A.T. came up with the concept of the study, performed analyses, and wrote the first draft of the manuscript. L.Z. combined the dataset. All authors revised the manuscript and provided critical feedback.

Data sharing statement: Due to the data sharing agreements between individual cohorts and ART-CC, the data collected for this study cannot be shared. Data are owned by the individual cohorts and those wishing to access these data should contact the individual cohorts, details of which are given in the appendix.

Funding: The ART-CC is funded by the US National Institute on Alcohol Abuse and Alcoholism (U01-AA026209). JACS is funded by National Institute for Health Research Senior Investigator award NF-SI-0611-10168. AT is funded by the Wellcome Trust under a Sir Henry Wellcome Postdoctoral Fellowship (222770/Z/21/Z).

Funding for the individual ART-CC cohorts included in this analysis was from Alberta Health, Gilead, ANRS (France REcherche Nord&Sud Sida-hiv Hépatites), the French Ministry of Health, the Austrian Agency for Health and Food Safety (AGES), Stichting HIV Monitoring, the Dutch Ministry of Health, Welfare and Sport through the Centre for Infectious Disease Control of the National Institute for Public Health and the Environment, the TP-HIV by the German Centre for Infection Research (DZIF) (NCT02149004), the Instituto de Salud Carlos III through the Red Temática de Investigación Cooperativa en Sida (RD06/006, RD12/0017/0018 and RD16/0002/0006) as part of the Plan Nacional I + D + i and co-financed by ISCIII-Subdirección General de Evaluación and the Fondo Europeo de Desarrollo Regional (FEDER), ViiV Healthcare, Preben og Anna Simonsens Fond, ANRS-Maladies infectieuses émergentes, Institut National de la Santé et de la Recherche Médicale (INSERM), BMS, Janssen, MSD, the US National Institute on Alcohol Abuse and Alcoholism (U01-AA026230), the Spanish Ministry of Health, the Swiss National Science Foundation (grant 33CS30_134277), CFAR Network of Integrated Clinical Systems (1R24 AI067039-1, P30-AI-027757), the US Department of Veterans Affairs, the US National Institute on Alcohol Abuse and Alcoholism (U01-AA026224, U01-AA026209, U24-AA020794), the VHA Office of Research and Development, US National Institute of Allergy and Infectious Diseases (Tennessee Center for AIDS Research: P30 AI110527).

### Conflicts of interest

N.P. has received grants unrelated to this study and paid to his institution from Gilead Sciences Hellas and ECDC. A.A. received fees and grants unrelated to this study from Gilead, Merck, AstraZeneca, GSK, Pfizer, Moderna, Janssen-Cilag, ViiV. G.B. has received consulting fee from MedIQ, and payments and honoraria from the University of Kentucky and StateServ, whilst G.B.'s institution has received funding from Merck, Eli Lily, Kaiser Permanente, and Amgen. MC's institution received research grants and expert opinion fees from Gilead, MSD, and Viiv. M.J.G. has received honoraria from ad hoc membership of national HIV advisory boards, Merck, Gilead, and ViiV. R.T. has received grant funding from Gilead unrelated to this work. CS has received honoraria from Gilead Sciences, ViiV Healthcare and Janssen-Cilag for Scientific Advisory Boards and for educational lectures. NO's institution has received funding from the Preben og Anne Simonsens Fond. J.V. has personal fees from Merck/MSD, Gilead, Pfizer, Astellas Pharma, Basilea, German Centre for Infection Research (DZIF), University Hospital Freiburg/Congress and Communication, Academy for Infectious Medicine, University Manchester, German Society for Infectious Diseases (DGI), Ärztekammer Nordrhein, University Hospital Aachen, Back Bay Strategies, German Society for Internal Medicine (DGIM) and grants from Merck/MSD, Gilead, Pfizer, Astellas Pharma, Basilea, German Centre for Infection Research (DZIF), German Federal Ministry of Education and Research (BMBF). M.V.dV. received grants from ViiV Heathcare, MSD, and Gilead outside the submitted work and all paid to his institution. M.V.dV. receives personal fees from ViiV Heathcare, Gilead, and MSD all paid to his institution outside the submitted work. F.B. has received travel grants and honoraria from ViiV Healthcare, Gilead, ViiV, Janssen, and MSD, and support for attending meetings from Gilead, Janssen, MSD, and ViiV Healthcare. A.T., L.Z., R.I., G.L., J.P.V., T.R.S., H.M.C., M.J.S., S.M.I. and J.A.C.S. report no conflicts of interest.

## Supplementary Material

Supplemental Digital Content
