# Supplementary material for: Care interruptions and mortality among adults in Europe and North America
Source: AIDS. 2024 May 14;38(10):1533–42. doi: 10.1097/QAD.0000000000003924 (PMC11239093; doi:10.1097/QAD.0000000000003924)
Supplement: Supplemental Digital Content [file aids-38-1533-s001.docx]

**Care interruptions and mortality among adults in Europe and North America: a collaborative analysis of cohort studies**

**SUPPLEMENTARY MATERIALS**

*Included cohorts*

The included cohorts were (AHIVCOS, AMACS, ATHENA, Alberta, Aquitaine, CBC, CoRIS, DHK, Frankfurt, ICONA, KP, PISCIS, SHCS, UAB, UW, VACH, VACS, Vanderbilt – see here for further information: <http://www.bristol.ac.uk/art-cc/whoswho/>).

*Sensitivity analyses*

In a sensitivity analysis of the main analysis using Cox regression (described in the Statistical analysis section of the manuscript), rather than excluding it, we included follow-up of PWH returning to care with suppressed viral loads in the interruption groups, as well as follow-up of PWH returning to care with unsuppressed viral loads. We also performed a sensitivity analysis dropping follow-up periods where people resumed care with CD4 counts ≥350 cells/mm3. Due to the high levels of missingness, in a separate sensitivity analysis we investigated using ART start CD4 cell count values rather than time-updating them at the time of restarting care, regardless of availability at care re-initiation. As follow-up time was on average much longer for PWH who did not experience interruptions, we restricted follow-up to 90 days and 180 days (separately) after ART initiation or re-engagement with care to compare mortality rates directly after start ART and after an interruption. We performed a sensitivity analysis restricted follow-up to 3-years, to account for the median follow-up time in the post-interruption follow-up group. We also included a sensitivity analysis dropping follow-up occurring after a person’s third care interruption, to investigate removing the effect of extreme multiple interrupters, and further to this we performed a separate sensitivity analysis categorising the number of interruptions as 0, 1, 2, and ≥3. In sensitivity analyses we also investigated defining care interruptions as gaps of ≥270 days and ≥545 days instead of ≥365 days. Finally, we investigated not re-including follow-up of people who died after being lost-to-follow-up.
