# Supplementary material for: Care interruptions and mortality among adults in Europe and North America
Source: AIDS. 2024 May 14;38(10):1533–42. doi: 10.1097/QAD.0000000000003924 (PMC11239093; doi:10.1097/QAD.0000000000003924)
Supplement: Supplemental Digital Content [file aids-38-1533-s002.doc]

Supplementary table 1: Sensitivity analyses of adjusted mortality hazard ratios* for interruption status (I) – no/pre interruption (N) and post interruption (P)

| **Analysis** | **I** | **Person-years of observation** | **Deaths** | **Crude mortality rate per 1000 person-years (95% CI)** | **Adjusted hazard ratio (95% CI)*** |
| --- | --- | --- | --- | --- | --- |
| **Main analysis** | N | 509408 | 5469 | 10.7 (10.5-11.0) | 1 (reference) |
|  | P | 26925 | 635 | 23.6 (21.8-25.5) | 1.72 (1.57-1.88) |
| **Include post-return-to-care follow-up with suppressed viral loads** | N | 509408 | 5469 | 10.7 (10.5-11.0) | 1 (reference) |
|  | P | 49652 | 852 | 17.2 (16.0-18.4) | 1.60 (1.48-1.73) |
| **Drop post-return-to-care follow-up with CD4 counts ≥350** | N | 509408 | 5469 | 10.7 (10.5-11.0) | 1 (reference) |
|  | P | 19564 | 579 | 29.6 (27.3-32.1) | 1.77 (1.61-1.94) |
| **Use ART start CD4 counts rather than time-updating** | N | 509408 | 5469 | 10.7 (10.5-11.0) | 1 (reference) |
|  | P | 26925 | 635 | 23.6 (21.8-25.5) | 1.65 (1.51-1.81) |
| **Limiting follow-up to the 3-months after ART start/care interruptions** | N | 21867 | 751 | 34.3 (32.0-36.9) | 1 (reference) |
|  | P | 2943 | 105 | 35.7 (29.5-43.2) | 1.17 (0.94-1.46) |
| **Limiting follow-up to the 6-months after ART start/care interruptions** | N | 44290 | 1148 | 25.9 (24.5-27.5) | 1 (reference) |
|  | P | 5810 | 173 | 29.8 (25.7-34.6) | 1.29 (1.09-1.54) |
| **Define care interruptions as gaps of ≥270 days** | N | 441799 | 4415 | 10.0 (9.7-10.3) | 1 (reference) |
|  | P | 86023 | 1387 | 16.1 (15.3-17.0) | 1.49 (1.40-1.60) |
| **Define care interruptions as gaps of ≥545 days** | N | 542589 | 5890 | 10.9 (10.6-11.1) | 1 (reference) |
|  | P | 14011 | 326 | 23.3 (20.9-25.9) | 1.67 (1.48-1.88) |

*Adjusted for sex, age at ART initiation/re-initiation, CD4 count at ART initiation/re-initiation, year of ART initiation/re-initiation, and HIV acquisition method, with hazards stratified by cohort.
